# The association between sarcopenia and the prognosis of patients with high tumor burden hepatocellular carcinoma receiving hepatic artery infusion chemotherapy combined with lenvatinib and programmed death receptor-1 inhibitor: a multicenter, propensity score matching comparative study

**DOI:** 10.3389/fimmu.2026.1782185

**Published:** 2026-08-18

**Authors:** Xinge Li, Yunlong Dong, Wen Chen, Yan Wang, Pengfei Sun, Xu Chang, Chunxue Wu, Dianzhe Wang

**Affiliations:** 1 Department of Radiation Oncology, Shandong Cancer Hospital and Institute, Shandong First Medical University and Shandong Academy of Medical Sciences, Jinan, Shandong, China; 2 Department of Thoracic Surgery, Tianjin Baodi Hospital, Baodi Hospital Affiliated to Tianjin Medical University, Tianjin, China; 3 Department of Interventional Therapy, Shandong Cancer Hospital and Institute, Shandong First Medical University and Shandong Academy of Medical Sciences, Jinan, Shandong, China; 4 Department of Interventional Therapy, The First Affiliated Hospital with Nanjing Medical University, Nanjing, Jiangsu, China; 5 Department of Interventional Treatment, Tianjin Cancer Hospital Airport Hospital, National Clinical Research Center for Cancer, Tianjin’s Clinical Research Center for Cancer, Tianjin, China; 6 Department of Hepatobiliary Surgery, Shandong Cancer Hospital and Institute, Shandong First Medical University and Shandong Academy of Medical Sciences, Jinan, Shandong, China; 7 Department of General Surgery, Qilu Hospital, Cheeloo College of Medicine, Shandong University, Jinan, Shandong, China; 8 Department of Radiology, Shandong Cancer Hospital and Institute, Shandong First Medical University and Shandong Academy of Medical Sciences, Jinan, Shandong, China

**Keywords:** HAIC, high tumor burden, lenvatinib, PD-1, sarcopenia

## Abstract

**Background and objective:**

Patients with hepatocellular carcinoma (HCC) and a high tumor burden, such as those presenting with a tumor diameter of ≥ 10 cm or/and major portal vein tumor thrombosis (PVTT), generally face a poor prognosis. Sarcopenia is common in this population. Triple therapy combining hepatic arterial infusion chemotherapy (HAIC), lenvatinib, and programmed death receptor-1 (PD-1) inhibitors has shown encouraging outcomes in these patients. This study investigated the association between sarcopenia and the prognosis in patients with high tumor burden HCC patients receiving this triple therapy.

**Methods:**

This study included 449 patients with high tumor burden HCC patients who received HAIC-LEN-PD1 triple therapy of from January 2022 to December 2024. Sarcopenia was assessed using the skeletal muscle index (SMI). Propensity score matching (PSM) was used to balance baseline characteristics between groups. Treatment efficacy was evaluated by overall survival (OS), progression-free survival (PFS), objective response rate (ORR), and disease control rate (DCR). Cox proportional hazards regression analyses were performed to identify independent factors associated with OS and PFS.

**Results:**

Totally 449 patients were analyzed, including 131 in the sarcopenia group and 318 in the non-sarcopenia group. After PSM(1:1), 131 patients were matched in each group. Median OS was 12.2 months in the sarcopenia group and 18.1 months in the non-sarcopenia group(hazard ratio [HR]: 1.84; *p* < 0.001). PFS was also shorter among patients with sarcopenia, with a median of 6.9 months compared with 9.9 months among those without sarcopenia (HR: 1.53; *p* = 0.002). ECOG PS, and sarcopenia were independent predictors for OS and PFS (*p* < 0.05).

**Conclusion:**

Sarcopenia was associated with an poor prognosis in patients with high tumor burden HCC patients who received HAIC-LEN-PD1 therapy.

## Introduction

Hepatocellular carcinoma (HCC) represents the most prevalent form of primary liver cancer, constituting roughly 70% to 85% of all liver cancer cases. It is the sixth most frequently diagnosed malignancy worldwide and is the third leading cause of cancer-related death (1–3). In China, chronic hepatitis B virus (HBV) infection remains the predominant cause of HCC. Despite advances in screening and prevention, many patients are diagnosed only after the disease HCC has progressed to a high tumor burden stage, such as tumor diameter ≥ 10 cm, unilobar portal vein tumor thrombosis (Vp3), or main trunk or bilobar portal vein thrombosis (Vp4). Clinical outcome data for this subgroup are limited, largely because their poor prognosis has frequently led to their exclusion from clinical trials. And this population has few limited effective treatment options. Currently, the combination of hepatic arterial infusion chemotherapy(HAIC) with targeted agents and immunotherapy has emerged as a promising treatment strategy for patients with high tumor burden HCC, and several studies have shown improved survival with this approach (4–6). Identifying standardized prognostic markers for these patients is therefore clinically important.

Several clinical prognostic indicators have been used to predict outcomes in HCC patients undergoing HAIC combined with targeted therapy and immunotherapy, including hematological indices, the Barcelona Clinic Liver Cancer (BCLC) staging system and the child-pugh score, among others (5). However, these markers primarily reflect disease-specific characteristics and fail to comprehensively capture patient’s overall physical condition (7). Previous studies have shown that the physical performance status also significantly affects prognosis in patients with cancer (8, 9). Therefore, markers reflecting physical performance status should also be considered when screening prognostic indicators for HCC.

Skeletal muscles are an important part of the body composition that reflects patients’ metabolic and nutritional status of the patient and correlates with prognosis across a wide range of diseases (10–12). Computed tomography(CT) has become an important noninvasive tool for evaluating skeletal muscle mass and density in clinical practice (13). Sarcopenia is a progressive disorder characterized by substantial loss of skeletal muscle mass and function (14), and it poses substantial health risks. Although standardized cutoff values for diagnosing sarcopenia remain debated, multiple studies have shown that sarcopenia is associated with unfavorable prognosis in patients with hepatocellular carcinoma, colorectal cancer, and lung cancers (15–17). However, current clinical studies of the association between sarcopenia and outcomes in HCC have mainly focused focus on surgical treatment, interventional therapy, immunotherapy, targeted therapy, or combined targeted therapy and immunotherapy (18–21). Evidence remains limited regarding the association between sarcopenia and prognosis in patients with HCC treated with HAIC combined with targeted therapy and immunotherapy especially among those with a high tumor burden. In the clinical context of China, this study aims to evaluate the prognostic impact of sarcopenia in patients with high tumor burden HCC undergoing triple therapy with HAIC, lenvatinib and programmed death receptor-1 inhibitors (HAIC-LEN-PD1).

## Materials and methods

### Patient data

Between January 2022 and December 2024, patients with high tumor burden HCC who received HAIC-LEN-PD1 triple therapy were identified and retrieved from four medical centers including Shandong Cancer Hospital and Institute (Center 1), Tianjin Cancer Hospital Airport Hospital (Center 2), Qilu Hospital of Shandong University (center 3), and The First Affiliated Hospital with Nanjing Medical University (center 4). HCC was diagnosed according to the histological or radiological criteria defined in the American Association for the Study of Liver Diseases (AASLD) guidelines. The inclusion criteria were as follows: (1) age ≥ 18 years old, (2) high tumor burden, defined as: tumor diameter ≥ 10 cm or/and the presence of Vp3 or Vp4; (3) Child-Pugh class A or B liver function; (4) an Eastern Cooperative Oncology Group performance status (ECOG PS) score 0-2; (5) patients who underwent CT assessment within one month before treatment, (6) receipt of at least two cycles of HAIC-LEN-PD-1 therapy. Patients were excluded if they had (1) incomplete clinical or radiological data, (2) history of other malignancies, (3) were lost to follow-up. This retrospective study was approved by the Ethics Committee of Shandong Cancer Hospital and Institute (SDTHEC2025012). Because the analysis was retrospective, the requirement for informed consent was waived.

### Clinical data collection

Baseline clinical characteristics of patients were collected before treatment, including age, sex, etiology, BCLC stage, ECOG-PS, Child-Pugh class, albumin(ALB), Albumin-Bilirubin grade(ALBI) and alpha-fetoprotein (AFP). Lesion number, maximum lesion diameter, portal vein tumor thrombosis(PVTT), and extrahepatic spread(EHS) were assessed on CT images obtained within one month before treatment.

### Treatment protocol

All participants received oral lenvatinib, at a dose based on body weight: 12 mg per day for patients weighing 60 kg or more, and 8 mg per day for those under 60 kg. PD-1 inhibitors were administered intravenously within one day following the HAIC procedure and were repeated every three weeks at the prescribed dose.

For the HAIC procedure, a catheter or microcatheter was inserted into the primary hepatic artery supplying the tumor. Chemotherapy was initiated within 2 days after catheter placement. The following agents were infused through the catheter or microcatheter: oxaliplatin (85 mg/m² over 0–2 hours on Day 1), leucovorin (400 mg/m² over 2–3 hours on Day 1), and 5-fluorouracil (400 mg/m² as a bolus at 3 hours, followed by 2400 mg/m² over 46 hours on Days 1 and 2). Upon completion of drug infusion, the catheter and sheath were withdrawn. HAIC sessions were conducted every three weeks, with patients receiving two to six cycles, after which maintenance therapy with lenvatinib and PD-1 inhibitors was continued.

Lenvatinib was initiated 3 to 5 days before the first HAIC session to assess patient tolerability. Dose adjustments for lenvatinib (to 8 or 4 mg per day, or 4 mg every other day) were permitted for drug-related toxicity. Because no significant efficacy differences have been observed among different PD-1 inhibitors, selection was primarily guided by patients’ financial considerations and insurance coverage. All treatment plans were developed through multidisciplinary team discussions among oncologists, surgeons, and radiologists.

### Imaging assessment

USing the most recent CT scans obtained within one month before initiation of HAIC-LEN-PD1 triple therapy, two radiologists independently assessed total skeletal muscle area at L3 vertebral level for each patient; discrepancies were resolved by a third experienced senior physician. CoreSlicer software (www.coreslicer.com) was utilized to identify and quantify skeletal muscle area, with CT threshold values for skeletal muscle ranging from -29 to 150 HU.

The Skeletal Muscle Index (SMI) is widely regarded as a reliable measure of skeletal muscle mass and an internationally recognized parameter for diagnosing sarcopenia (21). SMI is calculated by dividing the cross-sectional muscle area by the square of the patient’s height (22). Following the Japan Society of Hepatology guidelines for sarcopenia in liver disease (23), we defined sarcopenia using the recommended SMI cutoff values: < 42 cm²/m² for males and < 38 cm²/m² for females. Patients were categorized according to these thresholds for further analyses.

### Outcomes and tumor response

The primary endpoint was overall survival (OS), which was defined as the interval from the initiation of HAIC-LEN-PD1 therapy to death from any cause or the date of last follow-up. Secondary endpoints included progression-free survival (PFS), objective response rate (ORR), disease control rate (DCR), and adverse events (AEs). PFS was measured from the start of HAIC-LEN-PD1 treatment to the first occurrence of radiologic disease progression or HCC-related death. Tumor response was evaluated according to RECIST version 1.1 and classified as complete response (CR), partial response (PR), stable disease (SD), or progressive disease (PD). ORR was calculated as the percentage of patients achieving CR or PR, while DCR was calculated as the percentage achieving CR, PR, or SD. AEs were graded according to the National Cancer Institute Common Terminology Criteria for Adverse Events, version 5.0.

### Statistical analysis

All statistical analyses were performed using SPSS version 25.0 or R version 4.4.2. Continuous variables are reported as means with standard deviations (SDs), and categorical variables are summarized as frequencies and percentages. Continuous variables were compared using the t-test or Mann-Whitney U-test, as appropriate, and categorical variables were compared using the chi-square test or Fisher’s exact test. Survival outcomes were analyzed using Kaplan-Meier curves, with differences assessed by the log-rank test. Independent predictors of OS and PFS were using univariate and multivariate Cox proportional hazards models. To reduce potential confounding, 1:1 nearest-neighbor propensity score matching (PSM) was performed using the MatchIt package in R, with a caliper width of 0.2; the propensity model included age, sex, etiology, AFP level, Child-Pugh class, BCLC stage, extrahepatic spread (EHS), PVTT, tumor diameter, tumor number, and ECOG PS. Multivariable Cox sensitivity analyses additionally adjusting for participating center and PD-1 inhibitor type were performed before and after PSM. Restricted cubic spline Cox models evaluated continuous SMI, and exploratory Kaplan-Meier analyses assessed outcomes by participating center, PD-1 inhibitor type, and HAIC session number. A p-value of less than 0.05 was considered statistically significant.

## Results

### Patient characteristics

**Figure 1:**
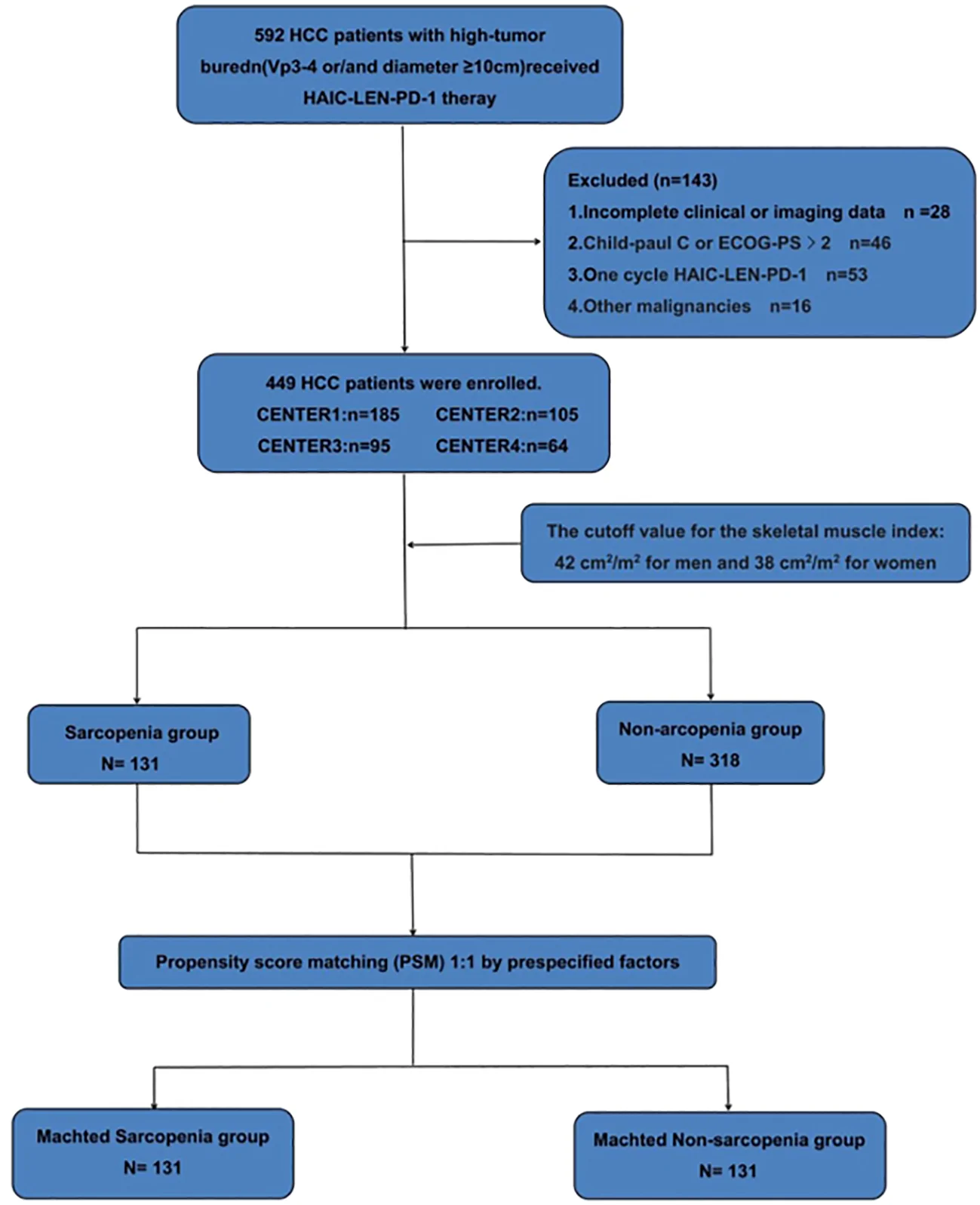
Flowchart of HCC patient selection.CENTER 1, Shandong Cancer Hospital and Institute; CENTER 2, Tianjin Cancer Hospital Airport Hospital; CENTER 3, Qilu Hospital of Shandong University; CENTER 4, The First Affiliated Hospital with Nanjing Medical University.

The patient selection process is illustrated in **Figure 1**. A total of 449 patients were enrolled in the study, including 131 individuals in the sarcopenia group and 318 in the non-sarcopenia group. After PSM, 131 patients were matched in each group. Baseline characteristics of all patients are shown in **Table 1**. The sarcopenia group included 26 females and 105 males, whereas the non-sarcopenia group included 24 females and 294 males (*p* < 0.001). Patients aged 60 years or older made up a larger percentage of the sarcopenia group than the non-sarcopenia group (42.0% vs. 26.1%, *p* < 0.001). EHS was observed in 47.3% of patients with sarcopenia compared with 31.4% of those without sarcopenia (*p* = 0.001). The non-sarcopenia group had a higher proportion of patients with an ECOG PS score of 0(80.2% vs. 63.4%, *p* < 0.001). Most patients in both groups had Child-Pugh class A liver function(sarcopenia, n = 94; non-sarcopenia, n = 256) and a history of hepatitis B (sarcopenia, n = 114; non-sarcopenia, n = 285), and these patients generally received antiviral therapy. The sarcopenia group included a higher proportion of patients with ALB < 35 g/L(29.0% vs. 17.3%, *p* = 0.005). Lenvatinib was the targeted therapeutic agent used in this study was lenvatinib, and the immune therapy drugs included camrelizumab (n = 207), sintilimab (n = 99), tislelizumab (n = 110), and other drugs (n = 33). Before matching, no significant between-group differences were observed in tumor diameter, tumor number, BCLC stage, ALBI grade or presence of PVTT(*p* > 0.05). PSM was performed to reduce selection bias. After PSM was carried out in this study. Following PSM, baseline characteristics were well balanced between the two groups (all *p* > 0.05; **Table 1**; **Supplementary Figure 3**).

**Table 1:** Baseline characteristics before and after PSM.

Characteristics	Primary cohort				PSM cohort			
	Sarcopenia N=131(%)	Non-sarcopenia N=318(%)	*P*	SMD	Sarcopenia N=131(%)	Non-sarcopenia N=131(%)	*P*	SMD
**Age**			<0.001	0.340			0.802	0.031
<60	76 (58.0)	235 (73.9)			76 (58.0)	78 (59.5)		
≥60	55 (42.0)	83 (26.1)			55 (42.0)	53 (40.5)		
**Sex**			<0.001	0.364			0.421	0.099
Female	26 (19.8)	24 (7.5)			26 (19.8)	21 (16.0)		
Male	105 (80.2)	294 (92.5)			105 (80.2)	110 (84.0)		
**ECOG PS**			<0.001	0.381			0.793	0.080
0	83 (63.4)	255 (80.2)			83 (63.4)	88 (67.2)		
1	28 (21.4)	43 (13.5)			28 (21.4)	26 (19.8)		
2	20 (15.3)	20 (6.3)			20 (15.3)	17 (13.0)		
**Etiology**			0.426	0.081			0.856	0.022
Others	17 (13.0)	33 (10.4)			17 (13.0)	18 (13.7)		
HBV	114 (87.0)	285 (89.6)			114 (87.0)	113 (86.3)		
**AFP**			0.340	0.100			0.424	0.099
≤400	44 (33.6)	122 (38.4)			44 (33.6)	38 (29.0)		
>400	87 (66.4)	196 (61.6)			87 (66.4)	93 (71.0)		
**Child-Pugh class**			0.042	0.206			0.785	0.034
A	94 (71.8)	256 (80.5)			94 (71.8)	92 (70.2)		
B	37 (28.2)	62 (19.5)			37 (28.2)	39 (29.8)		
**BCLC stage**			0.763	0.031			0.844	0.024
A/B	14 (10.7)	31 (9.7)			14 (10.7)	15 (11.5)		
C	117 (89.3)	287 (90.3)			117 (89.3)	116 (88.5)		
**EHS**			0.001	0.329			0.804	0.031
Absent	69 (52.7)	218 (68.6)			69 (52.7)	71 (54.2)		
Present	62 (47.3)	100 (31.4)			62 (47.3)	60 (45.8)		
**PVTT**			0.234	0.122			0.894	0.017
Vp1-2/ none	41 (31.3)	82 (25.8)			41 (31.3)	40 (30.5)		
Vp 3-4	90 (68.7)	236 (74.2)			90 (68.7)	91 (69.5)		
**Tumor diameter**			0.112	0.170			0.492	0.085
<10 cm	22 (16.8)	75 (23.6)			22 (16.8)	18 (13.7)		
≥10 cm	109 (83.2)	243 (76.4)			109 (83.2)	113 (86.3)		
**Tumor number**			0.990	0.001			0.766	0.037
Single	30 (22.9)	73 (23.0)			30 (22.9)	28 (21.4)		
Multiple	101 (77.1)	245 (77.0)			101 (77.1)	103 (78.6)		
**ALB**			0.005	0.280			0.784	0.034
≥35 g/L	93 (71.0)	263 (82.7)			93 (71.0)	95 (72.5)		
<35 g/L	38 (29.0)	55 (17.3)			38 (29.0)	36 (27.5)		
**ALBI grade**			0.111	0.167			0.701	0.047
1	47 (35.9)	140 (44.0)			47 (35.9)	50 (38.2)		
2/3	84 (64.1)	178 (56.0)			84 (64.1)	81 (61.8)		
**ICIs**			0.981				0.503	
Camrelizumab	61 (46.6)	146 (45.9)			61 (46.6)	56 (42.7)		
Tislelizumab	31 (23.7)	79 (24.8)			31 (23.7)	32 (24.4)		
Sintilimab	30 (22.9)	69 (21.7)			30 (22.9)	27 (20.6)		
Others	9 (6.9)	24 (7.5)			9 (6.9)	16 (12.2)		
**SMI**	37.34 ± 3.51	50.30 ± 6.54	<0.001		37.34 ± 3.51	49.14 ± 7.06	<0.001	

Bold values represent the baseline characteristics of the study participants.

### Prognosis

**Figure 2:**
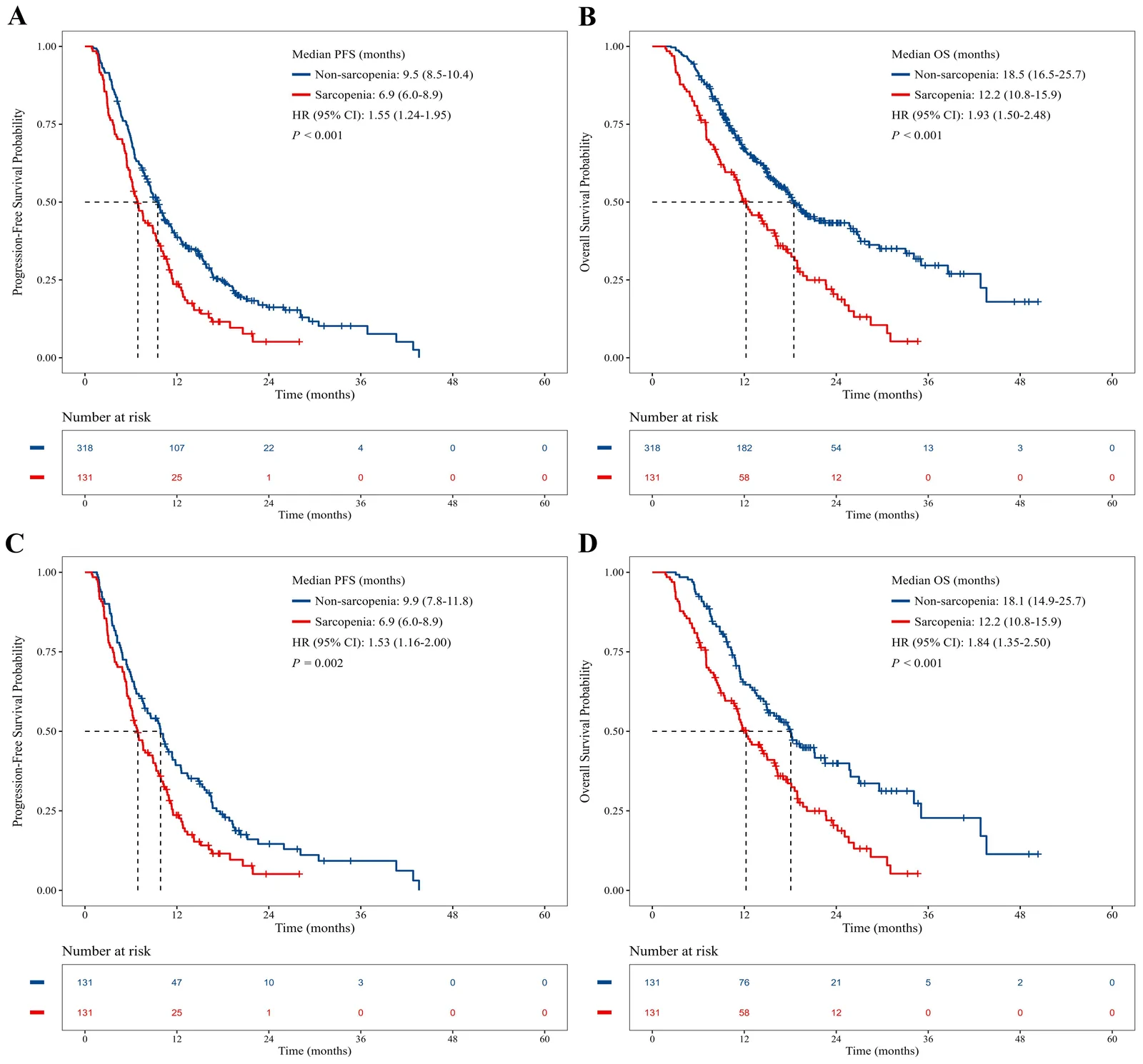
Kaplan-Meier curves of overall survival (OS) and progression-free survival (PFS) before and after propensity score matching(PSM). Kaplan-Meier curves of PFS **(A)** and OS **(B)** before PSM; Kaplan-Meier curves of PFS **(C)** and OS **(D)** for patients after PSM.

Before PSM, median PFS (mPFS) was 9.5 (95% CI: 8.5-10.4) months in the non-sarcopenia group and 6.9 (95% CI: 6.0-8.9) in the sarcopenia group(HR = 1.55, *p* < 0.001; **Figure 2A**). Similarly, median OS (mOS) was longer in the non-sarcopenia group than in the sarcopenia group (18.5 months [95% CI: 16.5-26.7] vs. 12.2 months [95% CI: 10.8-15.9]; HR = 1.93, *p* < 0.001; **Figure 2B**).

**Figure 3:**
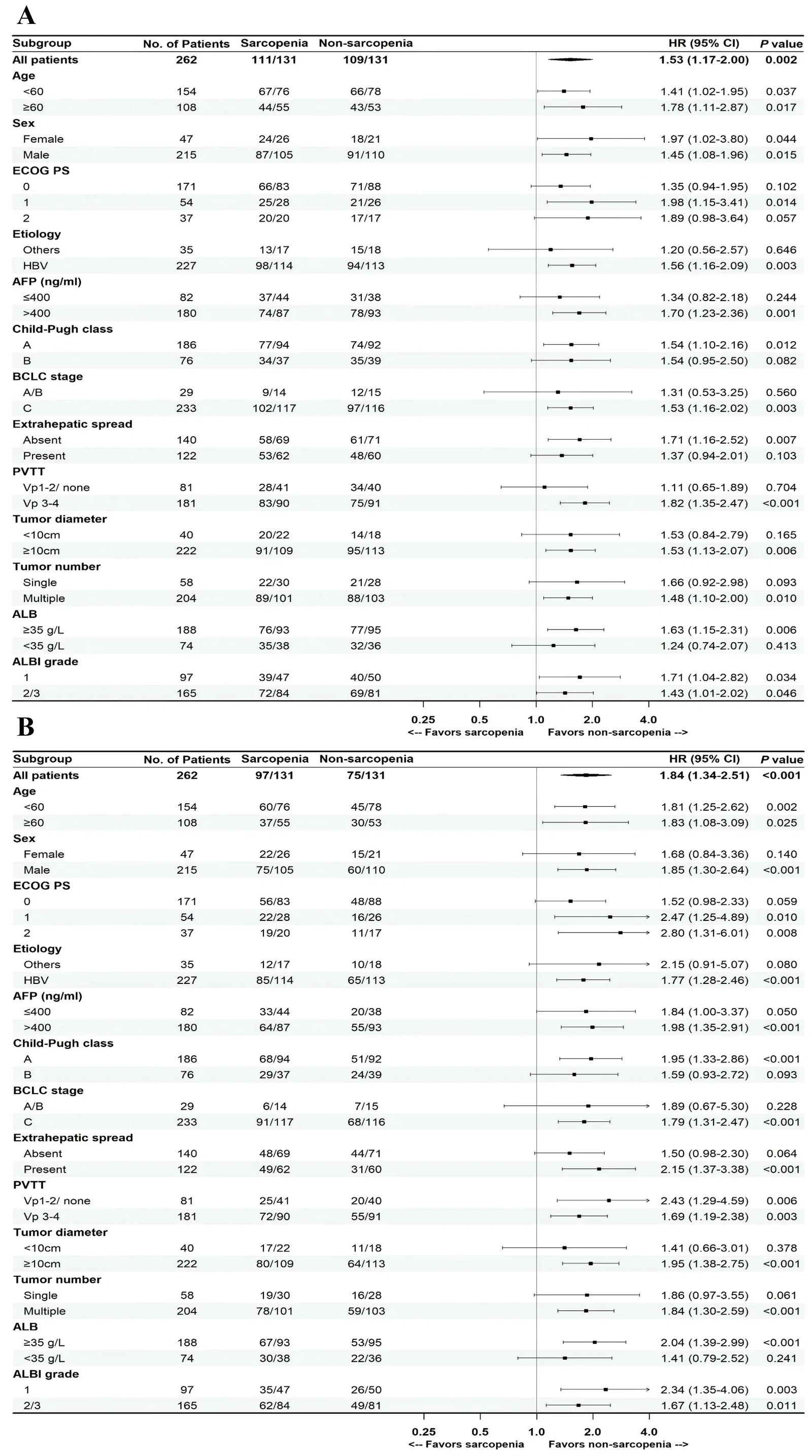
Subgroup analysis of progression-free survival(PFS) and overall survival(OS). Forest plots of OS **(A)** and PFS **(B)** in different patient subgroups after PSM.

After PSM, patients with sarcopenia continued to have inferior outcomes, with a mOS of 12.2 months compared with 18.1 months in the non-sarcopenia group (95% CI:1.35-2.50, HR = 1.84, *p* < 0.001) and a mPFS of 6.9 months compared with 9.9 months (95% CI:1.16-2.00, HR = 1.53, *p* = 0.002; **Figures 2D, C**). Further subgroup analysis shown in **Figure 3** and **Supplementary Figure 1** indicated that patients with sarcopenia may exhibit decreased mOS and mPFS in most subgroups compared with those without sarcopenia.

Exploratory analyses showed no significant PFS or OS differences across participating centers or PD-1 inhibitor types (**Supplementary Figures 2**, **4**), whereas patients receiving >4 HAIC sessions had longer PFS and OS (**Supplementary Figure 6**); continuous SMI was associated with both outcomes (**Supplementary Figure 5**).

### Prognostic predictors

Cox regression analysis was performed to identify factors associated with patient prognosis. Results of univariate and multivariate analyses for OS and PFS after PSM are presented in **Table 2**. Independent prognostic factors for OS included age, ECOG PS, tumor number, and sarcopenia. Sex, ECOG PS and sarcopenia were identified as independent predictors affecting PFS. **Supplementary Table 1** presents the Cox regression results before PSM, in which sarcopenia was also identified as an independent prognostic factor for both OS and PFS.

**Table 2:** Cox analyses associated with progression-free survival and overall survival after PSM.

Variables	Univariate analysis			Multivariate analysis		
	HR	95% CI	*P*	HR	95% CI	*P*
PFS analyses						
Sarcopenia (Present vs. Absent)	1.53	1.17-2.00	0.002	1.61	1.22-2.13	<0.001
Age (≥60 vs. <60)	0.65	0.50-0.85	0.002	0.68	0.51-0.89	0.005
Sex (Male vs. Female)	0.86	0.63-1.18	0.356			
Etiology (HBV vs. Others)	1.31	0.90-1.91	0.162			
AFP (>400 vs. ≤400)	1.12	0.84-1.48	0.448			
Child-Pugh class (B vs. A)	1.31	0.99-1.74	0.058			
BCLC stage (C vs. A/B)	1.54	1.00-2.37	0.048	1.28	0.84-1.95	0.253
EHS (Present vs. Absent)	1.16	0.88-1.52	0.301			
PVTT (Vp 3–4 vs. Vp1-2/ none)	1.30	0.98-1.72	0.069			
Tumor diameter (≥10 cm vs. <10 cm)	1.00	0.66-1.50	0.984			
Tumor number (Multiple vs. Single)	1.53	1.08-2.19	0.018	1.56	1.08-2.25	0.019
ECOG PS (1/2 vs. 0)	1.60	1.22-2.11	<0.001	1.78	1.24-2.55	0.002
ALB (<35 g/L vs. ≥35 g/L)	1.29	1.00-1.66	0.049	0.88	0.61-1.25	0.466
ALBI grade (2/3 vs. 1)	1.31	1.02-1.69	0.034	1.05	0.79-1.41	0.739
OS analyses						
Sarcopenia (Present vs. Absent)	1.84	1.34-2.51	<0.001	2.01	1.44-2.80	<0.001
Age (≥60 vs. <60)	0.83	0.62-1.11	0.200			
Sex (Male vs. Female)	0.56	0.39-0.81	0.002	0.49	0.34-0.72	<0.001
Etiology (HBV vs. Others)	1.06	0.71-1.58	0.786			
AFP (>400 vs. ≤400)	1.30	0.95-1.77	0.096			
Child-Pugh class (B vs. A)	1.25	0.89-1.74	0.191			
BCLC stage (C vs. A/B)	1.54	0.89-2.67	0.120			
EHS (Present vs. Absent)	1.14	0.84-1.54	0.394			
PVTT (Vp 3–4 vs. Vp1-2/ none)	1.26	0.91-1.73	0.157			
Tumor diameter (≥10 cm vs. <10 cm)	1.05	0.71-1.55	0.806			
Tumor number (Multiple vs. Single)	1.31	0.94-1.84	0.111			
ECOG PS (1/2 vs. 0)	1.95	1.45-2.63	<0.001	2.34	1.75-3.15	<0.001
ALB (<35 g/L vs. ≥35 g/L)	1.31	0.96-1.80	0.088			
ALBI grade (2/3 vs. 1)	1.26	0.96-1.65	0.095			

After adjustment for participating center and PD-1 inhibitor type, the association of sarcopenia with shorter PFS and OS remained consistent before and after PSM, while no statistically significant associations were observed for participating center or PD-1 inhibitor type (**Supplementary Tables 2**, **3**).

### Tumor response

**Table 3** summarizes tumor response. Based on RECIST version 1.1, no significant differences in ORR or DCR were observed between the sarcopenia and non-sarcopenia groups before and after propensity matching (*p* > 0.05).

**Table 3:** Tumor responses evaluated by RECIST 1.1 before and after PSM.

Before PSM	Response	Sarcopenia N=131(%)	Non-sarcopenia N=318(%)	*P*
	CR	0 (0.0)	1 (0.3)	
	PR	48 (36.6)	144 (45.3)	
	SD	67 (51.1)	139 (43.7)	
	PD	16 (12.2)	34 (10.7)	
	ORR (CR+PR)	48 (36.6)	145 (45.6)	0.081
	DCR (CR+PR+SD)	115 (87.8)	284 (89.3)	0.641
After PSM	Response	Sarcopenia N=131(%)	Non-sarcopenia N=131(%)	*P*
	CR	0 (0.0)	0 (0.0)	
	PR	48 (36.6)	53 (40.5)	
	SD	67 (51.1)	63 (48.1)	
	PD	16 (12.2)	15 (11.5)	
	ORR (CR+PR)	48 (36.6)	53 (40.5)	0.526
	DCR (CR+PR+SD)	115 (87.8)	116 (88.5)	0.848

### Adverse events

**Table 4** summarizes the AEs. The sarcopenia group had a higher overall incidence of AEs of any-grade than the non-sarcopenia group, including anaemia, fatigue, weight loss, vomiting, hyboalbuminaemia, and decreased appetite (*p* < 0.05). The frequency of grade 3–4 AEs was also higher in the sarcopenia group, including Anaemia, Weight loss, Vomiting, Abdominal pain, Hyboalbuminaemia (*p* < 0.05). No treatment-related deaths occurred, and all AEs were effectively managed.

**Table 4:** Adverse events after PSM.

Adverse event	Sarcopenia (n=131)		Non- sarcopenia (n=131)		*P*	
	Any grade	Grade 3-4	Any grade	Grade 3-4	Any grade	Grade 3-4
Neutropenia	51 (38.9%)	15 (11.5%)	47 (35.9%)	10(7.6%)	0.610	0.293
Anaemia	28 (21.4%)	14 (10.7%)	9 (6.9%)	1 (0.8%)	0.001	0.001
Thrombocytopenia	58 (44.3%)	12 (9.2%)	52 (39.7%)	9 (6.9%)	0.453	0.495
Fatigue	82 (62.6%)	14 (10.7%)	44 (33.6%)	8 (6.1%)	<0.001	0.181
Hypertension	63 (48.1%)	8 (6.1%)	68(51.9%)	7 (5.3%)	0.537	0.790
Weight loss	55 (42.0%)	14(10.7%)	26 (19.8%)	5 (3.8%)	<0.001	0.032
Hypothyroidism	40 (30.5%)	5 (3.8%)	38 (29.0%)	4 (3.1%)	0.787	0.734
Vomiting	33 (25.2%)	8 (6.1%)	7(5.3%)	1 (0.8%)	<0.001	0.042
Diarrhea	10 (7.6%)	3 (2.3%)	10 (7.6%)	2 (1.5%)	1	0.652
Abdominal pain	40 (30.5%)	9(6.9%)	36(27.5%)	2 (1.5%)	0.586	0.031
Elevated ALT	71 (54.4%)	23 (17.6%)	68(51.9%)	16(12.2%)	0.710	0.224
Elevated AST	72 (54.2%)	24 (18.3%)	65 (49.6%)	15 (11.5%)	0.387	0.118
Hyperbilirubinacemia	51(38.9%)	13(9.9%)	50(38.2%)	8 (6.1%)	0.899	0.255
Hyboalbuminaemia	68 (51.9%)	25 (19.1%)	37 (28.2%)	7 (5.3%)	<0.001	0.001
Decreased appetite	78 (59.5%)	17(13.0%)	40(30.5%)	9(6.9%)	<0.001	0.098
Elevated creatinine	14(10.7%)	3 (2.3%)	6 (4.6%)	1 (0.8%)	0.063	0.314
Immune-related hepatitis	6(4.6%)	1 (0.8%)	6 (4.6%)	1 (0.8%)	1	1
Immune-related pneumonitis	4 (3.1%)	1 (0.8%)	2 (1.5%)	0 (0.0%)	0.409	1
Immune-related dermatitis	5 (3.8%)	0 (0%)	2 (1.5%)	0 (0.0%)	0.250	–
Immune-related myocarditis	1 (0.8%)	0 (0%)	1 (0.8%)	0 (0.0%)	1	–

## Discussion

High tumor burden is frequently observed at initial diagnosis among Chinese patients with HCC and is associated with poor prognosis. Recent evidence suggests that combining HAIC with targeted therapy and immunotherapy yields encouraging outcomes in patients with advanced HCC (4–6). This study evaluated the association between baseline muscle status and prognosis in patients receiving HAIC-LEN-PD-1 therapy. We analyzed a cohort of 449 patients with high tumor burden HCC treated with HAIC-LEN-PD-1 triple therapy and confirmed that sarcopenia was a predictor of poor prognosis in this population. Before PSM, both mOS and mPFS were significantly shorter in the sarcopenia group than in the non-sarcopenia group (mOS: 12.2 vs. 18.5 months, *p* < 0.001; mPFS: 6.9 vs. 9.5 months, *p* < 0.001). These differences persisted after matching, with the sarcopenia group continuing to have shorter mOS (12.2 vs. 18.1 months, *p* < 0.001) and mPFS (6.9 vs. 9.9 months, *p* = 0.002).

The occurrence and development of sarcopenia is multifactorial, involving malnutrition, metabolic disturbances, catabolic states, hormonal deficiencies, and changes in cytokine levels (24, 25). Sarcopenia serves as an indirect indicator of overall health status (26). Because malignant tumors commonly lead to cachexia, the relationship between cancer and sarcopenia has been actively explored. One study showed that CT-diagnosed preoperative sarcopenia can predict major complications and prognosis after NSCLC resection (17). In addition, a meta-analysis of colorectal cancer without distant metastasis, which included 13 studies and 6, 600 patients, showed that both preoperative and postoperative sarcopenia were associated with poorer OS and PFS (27). The association between HCC and sarcopenia remains an active area of research. A study of patients undergoing liver transplantation found that muscle loss >14.2% after transplantation was an independent predictor of decreased OS and PFS (28). Liu et al. demonstrated that progressive muscle loss and myosteatosis were significant prognostic indicators of poorer outcomes in patients with advanced liver cancer undergoing immunotherapy (29). Similarly, Sun et al. observed that patients with HCC and pre-existing sarcopenia who received lenvatinib and PD-1 inhibitors had worse survival outcomes and a higher rate of adverse events (20). In patients with HCC, treated with interventional therapy combined with targeted agents and immunotherapy, an increase in skeletal muscle mass after treatment has been shown to predict favorable OS (30). Most current research on HCC and sarcopenia has focused on patients receiving monotherapy or dual therapy. Although a few studies have evaluated regimens combining interventional therapy, targeted therapy, and immunotherapy, the local treatment modalities were limited to transarterial chemoembolization (TACE) or included both HAIC and TACE. Given the encouraging outcomes associated with HAIC combined with targeted therapy and immunotherapy in advanced HCC, this study specifically focuses on patients treated with HAIC-LEN-PD-1 triple therapy.

The efficacy of HCC treatment varies substantially according to tumor burden. Because high tumor burden is common among Chinese patients at initial diagnosis and is a major clinical focus in HCC treatment, stratified studies of this subgroup are needed. Portal vein tumor thrombus is present in 10-40% of patients with HCC, and is associated with poor prognosis, with median survival ranging from only 2.7 to 4.0 months (31). Golfieri et al. found that, compared with small HCC lesions, large HCC lesions (>5 cm) had a significantly lower complete remission rate (25% vs. 64%) (32). Theoretically, greater tumor burden, imposes a greater burden on patients and leads to worse therapeutic outcomes, consistent with the findings of the aforementioned study. Given the characteristic of high-tumor burden in Chinese patients at initial diagnosis and the clinical focus on the treatment of high-tumor burden HCC, we think it is necessary to conduct stratified studies on patients with high-tumor burden.

Previous researchers recommended PSM for comparing clinical outcomes between eastern and western cohorts of patients undergoing HCC resection who were affected by sarcopenia (33). Drawing on their experience, PSM was applied to the cohort to investigate the association between sarcopenia and prognosis in patients with HCC in this HCC cohort. we performed 1:1 PSM to reduce baseline imbalance between groups, which reduced the sample size from 449 to 262 patients. Although matching improved internal balance, the loss of nearly half of the original cohort reduced statistical power and limited external validity. The matched subset may not fully represent the overall population of patients with high tumor burden HCC, and residual unmeasured confounding cannot be completely excluded even after PSM adjustment.

This study showed that patients in the sarcopenia group had shorter OS and PFS than those in the non-sarcopenia group, and multivariate Cox regression analysis identified sarcopenia as an independent risk factor for both OS and PFS. These findings are consistent with most previous studies (34). In patients with HCC, changes in muscle status result from the interplay of multiple factors, including malnutrition, impaired liver function, hormonal and cytokine imbalances, as well as immune system disturbances, all of which contribute to muscle loss. Diminished muscle mass may also adversely affect treatment efficacy (29). Similarly, research has shown that skeletal muscle depletion can impair host immune competence (35), which may help explains the strong association between sarcopenia and adverse outcomes.

Our study demonstrated that there were no statistically significant differences in ORR or DCR between groups, and the survival difference may primarily reflect host factors such as frailty or systemic disease burden. However, previous studies have not yielded consistent results regarding tumor response (36, 37), and further investigation is still needed. Regarding AEs, both the sarcopenia and non-sarcopenia groups experienced similar types of events. We agree that some reported AEs, particularly hypoalbuminemia, weight loss, and decreased appetite, may reflect a combination of treatment-emergent toxicity, baseline nutritional status, liver reserve, and disease-related deterioration. Some AEs with high incidence rates, such as vomiting, abdominal pain, and decreased blood cell counts, are primarily related to the cytotoxic effects of chemotherapeutic agents on hematopoietic cells and gastrointestinal cells. Both groups of patients had poor liver function, which was related not only to the cytotoxic effects of chemotherapeutic agents but may also be attributable to portal vein blood flow obstruction caused by PVTT. However, these AEs were all effectively managed. Following HAIC completion, elevated ALT, AST, and TBIL typically returned to normal ranges within 1 week.

There are several limitations to this study. To begin with, as a retrospective analysis, even though PSM was utilized to enhance the comparability of baseline characteristics, some degree of selection bias and residual confounding may still persist. Second, this study only enrolled Chinese patients with high-tumor burden HCC; therefore, whether these findings can be generalized to other ethnic or geographic populations remains to be validated in independent cohorts. Third, although SMI is a recognized standard for assessing sarcopenia, there is no consensus on the cutoff value for SMI. This study used the SMI cutoff value determined by the Japanese Society of Hepatology (23), and supplementary analyses using SMI as a continuous variable were performed to assess the robustness of our findings (**Supplementary Figure 5**). Fourth, as a retrospective study, even though we carefully reviewed the medical records, standardized collection of some clinical and treatment-related indicators remains challenging. Fifth, treatment heterogeneity was unavoidable in this real-world multicenter cohort, as the selection of PD-1 inhibitors and treatment exposure were not protocol mandated (**Supplementary Figures 2**, **6**). Lastly, although subgroup analyses were performed to examine the consistency of the findings, these analyses were exploratory and some subgroups included a limited number of patients, which reduced the statistical power to detect potential interaction effects or subgroup-specific heterogeneity.

## Conclusion

Sarcopenia is a negative prognostic predictor for HCC patients with high tumor burden receiving HAIC-LEN-PD1 therapy.

## Data Availability

The raw data supporting the conclusions of this article will be made available by the authors, without undue reservation.
