# Supplementary material for: The association between sarcopenia and the prognosis of patients with high tumor burden hepatocellular carcinoma receiving hepatic artery infusion chemotherapy combined with lenvatinib and programmed death receptor-1 inhibitor: a multicenter, propensity score matching comparative study
Source: Front Immunol. 2026 Aug 18;17:1782185. doi: 10.3389/fimmu.2026.1782185 (PMC13529962; doi:10.3389/fimmu.2026.1782185)
Supplement: Supplementary file 1 [file Table1.docx]

**Table S1. Cox analyses associated with progression-free survival and overall survival before PSM.**

| **Variables** | **Univariate Analysis** | | | **Multivariate Analysis** | | |
| --- | --- | --- | --- | --- | --- | --- |
|  | **HR** | **95% CI** | ***P*** | **HR** | **95% CI** | ***P*** |
| **PFS analyses** |  |  |  |  |  |  |
| Sarcopenia (Present vs. Absent) | 1.55 | 1.24-1.95 | <0.001 | 1.50 | 1.19-1.89 | <0.001 |
| Age (≥60 vs. <60) | 0.81 | 0.65-1.02 | 0.073 |  |  |  |
| Sex (Male vs. Female) | 0.80 | 0.58-1.10 | 0.169 |  |  |  |
| Etiology (HBV vs. Others) | 1.12 | 0.80-1.55 | 0.515 |  |  |  |
| AFP (>400 vs. ≤400) | 1.14 | 0.92-1.41 | 0.231 |  |  |  |
| Child-Pugh class (B vs. A) | 1.41 | 1.11-1.80 | 0.005 | 1.09 | 0.81-1.46 | 0.581 |
| BCLC stage (C vs. A/B) | 1.47 | 1.02-2.13 | 0.040 | 1.45 | 1.00-2.11 | 0.047 |
| EHS (Present vs. Absent) | 1.19 | 0.96-1.47 | 0.121 |  |  |  |
| PVTT (Present vs. Absent) | 1.13 | 0.89-1.43 | 0.317 |  |  |  |
| Tumor diameter (≥10 cm vs. <10 cm) | 1.27 | 0.98-1.65 | 0.065 |  |  |  |
| Tumor number (Multiple vs. Single) | 1.31 | 1.02-1.69 | 0.037 | 1.29 | 1.00-1.67 | 0.049 |
| ECOG PS (1/2 vs. 0) | 1.63 | 1.29-2.06 | <0.001 | 1.52 | 1.15-2.01 | 0.004 |
| ALB (<35 g/L vs. ≥35 g/L) | 1.19 | 0.92-1.53 | 0.179 |  |  |  |
| ALBI grade (2/3 vs. 1) | 1.25 | 1.01-1.54 | 0.040 | 0.99 | 0.77-1.26 | 0.910 |
| **OS analyses** |  |  |  |  |  |  |
| Sarcopenia (Present vs. Absent) | 1.93 | 1.50-2.48 | <0.001 | 1.85 | 1.42-2.39 | <0.001 |
| Age (≥60 vs. <60) | 0.99 | 0.76-1.28 | 0.927 |  |  |  |
| Sex (Male vs. Female) | 0.50 | 0.36-0.71 | <0.001 | 0.54 | 0.38-0.78 | <0.001 |
| Etiology (HBV vs. Others) | 0.99 | 0.67-1.46 | 0.956 |  |  |  |
| AFP (>400 vs. ≤400) | 1.41 | 1.09-1.82 | 0.009 | 1.23 | 0.94-1.61 | 0.139 |
| Child-Pugh class (B vs. A) | 1.57 | 1.19-2.07 | 0.001 | 1.02 | 0.72-1.46 | 0.894 |
| BCLC stage (C vs. A/B) | 1.92 | 1.16-3.19 | 0.011 | 1.32 | 0.73-2.38 | 0.358 |
| EHS (Present vs. Absent) | 1.35 | 1.05-1.73 | 0.019 | 1.23 | 0.94-1.62 | 0.137 |
| PVTT (Present vs. Absent) | 1.35 | 1.01-1.79 | 0.042 | 1.33 | 0.95-1.86 | 0.098 |
| Tumor diameter (≥10 cm vs. <10 cm) | 1.19 | 0.89-1.59 | 0.252 |  |  |  |
| Tumor number (Multiple vs. Single) | 1.12 | 0.84-1.49 | 0.458 |  |  |  |
| ECOG PS (1/2 vs. 0) | 2.11 | 1.62-2.75 | <0.001 | 2.43 | 1.71-3.45 | <0.001 |
| ALB (<35 g/L vs. ≥35 g/L) | 1.38 | 1.03-1.84 | 0.029 | 0.64 | 0.43-0.95 | 0.025 |
| ALBI grade (2/3 vs. 1) | 1.51 | 1.17-1.93 | 0.001 | 1.22 | 0.91-1.64 | 0.178 |

## **Table S2. Full center- and PD-1 inhibitor-adjusted Cox models before PSM.**

| **Variables** | **PFS analysis** | | | **OS analysis** | | |
| --- | --- | --- | --- | --- | --- | --- |
|  | **HR** | **95% CI** | **P** | **HR** | **95% CI** | **P** |
| **Sarcopenia (Sarcopenia vs Non-sarcopenia)** | **1.42** | **1.12-1.81** | **0.004** | **1.77** | **1.35-2.33** | **<0.001** |
| Child-Pugh class (B vs A) | 1.10 | 0.82-1.48 | 0.525 | 1.01 | 0.71-1.43 | 0.976 |
| BCLC stage (C vs A/B) | 1.56 | 1.07-2.28 | 0.022 | 1.37 | 0.75-2.48 | 0.303 |
| Tumor number (Multiple vs Single) | 1.26 | 0.97-1.64 | 0.078 |  |  |  |
| ECOG PS (1/2 vs 0) | 1.53 | 1.16-2.04 | 0.003 | 2.44 | 1.70-3.49 | <0.001 |
| ALBI grade (2/3 vs 1) | 0.99 | 0.78-1.27 | 0.955 | 1.22 | 0.91-1.64 | 0.189 |
| Participating center (Center 2 vs Center 1) | 0.91 | 0.69-1.20 | 0.501 | 1.01 | 0.73-1.40 | 0.958 |
| Participating center (Center 3 vs Center 1) | 1.21 | 0.91-1.62 | 0.191 | 1.12 | 0.79-1.59 | 0.521 |
| Participating center (Center 4 vs Center 1) | 1.20 | 0.85-1.69 | 0.298 | 1.18 | 0.77-1.80 | 0.459 |
| PD-1 inhibitor (Tislelizumab vs Camrelizumab) | 1.03 | 0.78-1.34 | 0.857 | 0.88 | 0.64-1.21 | 0.442 |
| PD-1 inhibitor (Sintilimab vs Camrelizumab) | 0.93 | 0.70-1.22 | 0.599 | 0.90 | 0.64-1.25 | 0.516 |
| PD-1 inhibitor (Others vs Camrelizumab) | 1.00 | 0.65-1.53 | 0.984 | 1.20 | 0.74-1.97 | 0.457 |
| Sex (Male vs Female) |  |  |  | 0.54 | 0.38-0.78 | <0.001 |
| AFP (>400 vs <=400 ng/mL) |  |  |  | 1.23 | 0.94-1.62 | 0.137 |
| EHS (Present vs Absent) |  |  |  | 1.24 | 0.94-1.64 | 0.123 |
| PVTT (Present vs Absent) |  |  |  | 1.39 | 0.98-1.97 | 0.069 |
| ALB (<35 vs >=35 g/L) |  |  |  | 0.65 | 0.44-0.97 | 0.037 |

## **Table S3. Full center- and PD-1 inhibitor-adjusted Cox models after PSM.**

| **Variables** | **PFS analysis** | | | **OS analysis** | | |
| --- | --- | --- | --- | --- | --- | --- |
|  | **HR** | **95% CI** | **P** | **HR** | **95% CI** | **P** |
| **Sarcopenia (Sarcopenia vs Non-sarcopenia)** | **1.42** | **1.12-1.81** | **0.004** | **1.77** | **1.35-2.33** | **<0.001** |
| Child-Pugh class (B vs A) | 1.10 | 0.82-1.48 | 0.525 | 1.01 | 0.71-1.43 | 0.976 |
| BCLC stage (C vs A/B) | 1.56 | 1.07-2.28 | 0.022 | 1.37 | 0.75-2.48 | 0.303 |
| Tumor number (Multiple vs Single) | 1.26 | 0.97-1.64 | 0.078 |  |  |  |
| ECOG PS (1/2 vs 0) | 1.53 | 1.16-2.04 | 0.003 | 2.44 | 1.70-3.49 | <0.001 |
| ALBI grade (2/3 vs 1) | 0.99 | 0.78-1.27 | 0.955 | 1.22 | 0.91-1.64 | 0.189 |
| Participating center (Center 2 vs Center 1) | 0.91 | 0.69-1.20 | 0.501 | 1.01 | 0.73-1.40 | 0.958 |
| Participating center (Center 3 vs Center 1) | 1.21 | 0.91-1.62 | 0.191 | 1.12 | 0.79-1.59 | 0.521 |
| Participating center (Center 4 vs Center 1) | 1.20 | 0.85-1.69 | 0.298 | 1.18 | 0.77-1.80 | 0.459 |
| PD-1 inhibitor (Tislelizumab vs Camrelizumab) | 1.03 | 0.78-1.34 | 0.857 | 0.88 | 0.64-1.21 | 0.442 |
| PD-1 inhibitor (Sintilimab vs Camrelizumab) | 0.93 | 0.70-1.22 | 0.599 | 0.90 | 0.64-1.25 | 0.516 |
| PD-1 inhibitor (Others vs Camrelizumab) | 1.00 | 0.65-1.53 | 0.984 | 1.20 | 0.74-1.97 | 0.457 |
| Sex (Male vs Female) |  |  |  | 0.54 | 0.38-0.78 | <0.001 |
| AFP (>400 vs <=400 ng/mL) |  |  |  | 1.23 | 0.94-1.62 | 0.137 |
| EHS (Present vs Absent) |  |  |  | 1.24 | 0.94-1.64 | 0.123 |
| PVTT (Present vs Absent) |  |  |  | 1.39 | 0.98-1.97 | 0.069 |
| ALB (<35 vs >=35 g/L) |  |  |  | 0.65 | 0.44-0.97 | 0.037 |

**Figure S1.** Subgroup analysis of PFS and OS.Forest plots of overall survival(A) and progression-free survival(B) in different patient subgroups before PSM.

**Figure S2.** Kaplan-Meier curves of overall survival (OS) and progression-free survival (PFS) of programmed death receptor-1 inhibitors. Kaplan-Meier curves of PFS (A) and OS (B) for patients before propensity score matching (PSM); Kaplan-Meier curves of PFS (C) and OS (D) for patients after PSM.

**Figure S3.** Propensity score visualization plot

**Figure S4.** Kaplan-Meier curves of overall survival (OS) and progression-free survival (PFS) of multiple centers. Kaplan-Meier curves of PFS (A) and OS (B) for patients before propensity score matching (PSM); Kaplan-Meier curves of PFS (C) and OS (D) for patients after PSM.

**Figure S5.** Restricted cubic spline analyses evaluating the nonlinear associations of skeletal muscle index (SMI) with progression-free survival (PFS) and overall survival (OS). (A) Adjusted hazard ratios for PFS as a function of continuous SMI. The overall association P = 0.001, nonlinearity P = 0.132. (B) Adjusted hazard ratios for OS as a function of continuous SMI. The overall association P < 0.001, nonlinearity P = 0.002.

**Figure S6.** Kaplan-Meier curves of overall survival (OS) and progression-free survival (PFS) of the two groups of different HAIC sessions. Kaplan-Meier curves of PFS (A) and OS (B) for patients before propensity score matching (PSM); Kaplan-Meier curves of PFS (C) and OS (D) for patients after PSM.
